# The Role of Sterile Inflammation in Thrombosis: Consequences for Cardiovascular Disease and COVID-19

**DOI:** 10.1155/mi/8054886

**Published:** 2025-11-19

**Authors:** Gausal Azam Khan, Mashael Huwaikem, Kaustav Chowdhury, Humood Fahm Albugami, Arjun Ghosh

**Affiliations:** ^1^Department of Clinical Nutrition, College of Applied Medical Sciences, King Faisal University, Al Ahsa 31982, Saudi Arabia; ^2^Department of Rheumatology, All India Institute of Medical Science, Delhi, India; ^3^Department of Public Health, College of Applied Medical Sciences, King Faisal University, Al Ahsa, Saudi Arabia; ^4^Department of Biotechnology, Brainware University, Kolkata, India

**Keywords:** Covid-19, damage associated molecular patterns, pattern recognition receptors, sterile inflammation, thrombotic disorder

## Abstract

Sterile inflammation (SI) is an inflammatory response triggered by the release of damage-associated molecular patterns (DAMPs) from dying cells, distinct from normal inflammation in its origin from tissue injury and necrosis rather than microbial invasion. Circulating nucleic acids (CNAs), high-mobility group box 1 (HMGB1), von Willebrand factor (vWF), and S100b protein are notable markers of SI, indicative of tissue damage and implicated in thrombotic disorders. Innate immunity, involving cells like macrophages and dendritic cells, recognizes DAMPs via pattern recognition receptors (PRRs) like Toll-like receptors and NOD-like receptors, initiating inflammatory signaling cascades central to SI and its cardiovascular consequences. Thrombosis, a common outcome of SI, underscores the intricate interplay between inflammation and hemostasis, with hypoxia exacerbating thrombotic risk through platelet activation and endothelial dysfunction. The established link between inflammation and thrombosis highlights the clinical significance of SI, where molecules like HMGB1, extracellular RNA (eRNA), and eDNA actively participate in thromboembolic disorders. SI's relevance is particularly evident in COVID-19-induced thrombotic disorders, where dysregulated immune responses and endothelial dysfunction contribute to systemic inflammation and heightened thrombotic risk. Understanding SI's mechanisms in these contexts is vital for developing targeted therapies to mitigate vascular complications and enhance patient outcomes in cardiovascular diseases and COVID-19-associated thrombosis.

## 1. Introduction

The innate immune system is the first line of defense against harmful stimuli, recognizing pathogen-associated molecular patterns (PAMPs) and damage-associated molecular patterns (DAMPs) through pattern recognition receptors (PRRs) on immune cells. While inflammation typically results from microbial infections, sterile inflammation (SI) occurs in the absence of pathogens, triggered by tissue injury, ischemia-reperfusion injury, or chronic conditions such as atherosclerosis and particle-induced lung diseases [1]. This SI is driven by factors like circulating nucleic acids (CNAs), S100b, high-mobility group box 1 (HMGB1), and von Willebrand factor (vWF), which reflect tissue damage and contribute to thrombotic disorders, including the formation of blood clots. The interplay between inflammation and thrombosis is particularly relevant in cardiovascular pathology [2, 3], where SI can initiate and aggravate thrombotic events. In the context of COVID-19, SI plays a entral role in both acute and long-term complications. During severe infection, an exaggerated immune response or cytokine storm can lead to significant tissue damage, including conditions like acute respiratory distress syndrome (ARDS) [4, 5], while in long COVID-19, which is characterized by insoluble fibrin amyloid microclots that trap inflammatory molecules and prothrombotic proteins, these trapped molecules contribute to impaired fibrinolysis and hyperactivation, which may explain chronic symptoms like fatigue and cognitive impairment, highlighting the critical role of inflammation in long COVID-19 [6]. The aberrant immune response and endothelial dysfunction which could be seen in COVID-19 not only lead to systemic inflammation, but also increases the tendency for the increase of blood clots. Substantial evidence has also been known in demonstrating that DAMPs-induced inflammation is linked with various cardiometabolic diseases such as diabetes, NAFLD, ischemia-reperfusion injury, atherosclerosis, and cardiac pressure overload [7, 8]. This complex immune response and its consequences for thrombosis highlight the need to understand the mechanisms of SI to develop targeted therapies aimed at reducing vascular complications and improving patient outcomes in both cardiovascular diseases and COVID-19-related thrombosis.

If one acknowledges the causes of SI in such pathological conditions, one could eventually bring up more effective medications and treatments.

## 2. SI and Its Role in Innate Immune Activation

Inflammation is the body's natural response to harmful stimuli, which might include pathogens, irritants, or damaged cells. This reaction plays a major role in the defense mechanism of the body by removing injurious stimuli and initiating repair processes [9]. There are two major types of inflammation: acute and chronic based on severity [10]. Inflammation can also be classified as sterile and nonsterile. In SI, the immune response is oriented toward nonmicrobial antigens: drugs, burns, cancer, or autoimmune diseases. However, in non-SI, it refers to an inflammatory response that occurs in the body due to factors other than infection by microorganisms. Unlike SI, which is typically a response to tissue injury, trauma, or physical stress, non-SI often arises from conditions like autoimmune diseases, chronic diseases, or even environmental factors. In non-SI, the immune system responds to “danger signals” (such as damaged cells or irritants) but not directly to pathogens. These signals can come from internal sources, such as self-antigens in autoimmune diseases, or external sources like allergens, pollutants, or certain chemicals. The inflammation process involves the activation of immune cells, the release of cytokines, and the recruitment of inflammatory mediators like prostaglandins and leukotrienes.

Cytokine plays a very important and crucial role in maintaining health and homeostasis by regulating immune system. Like the stress response, inflammation is necessary for survival, but must be in controlled manner otherwise conditions like allergies, autoimmunity, chronic infections, and metabolic disorders can result due to improper regulation of inflammation. Stress hormones can either protect against or promote inflammation, depending on the context [11, 12]. Dysregulation of cytokine balance contributes to diseases like obesity, depression, and atherosclerosis [13, 14]. Complex cellular and inflammatory interactions are involved in the progress of vascular diseases. Endothelial cells, upon exposure to cytokines, undergo profound alterations of function that involve gene expression and de novo protein synthesis. The functional reprogramming of endothelial cells by cytokines is of importance especially in patients with chronic vascular inflammation. Several cytokines including TNF-α, TGF-β, and different interleukins (ILs) such as IL-1, IL-4, IL-6, IL-8, and IL-18 are involved in the development of various inflammatory cardiac pathologies, namely, ischemic heart disease, myocardial infarction, heart failure, and cardiomyopathies. In ischemia-related pathologies, most of the cytokines are released into circulation and serve as biological markers of inflammation [15].

**Figure 1:**
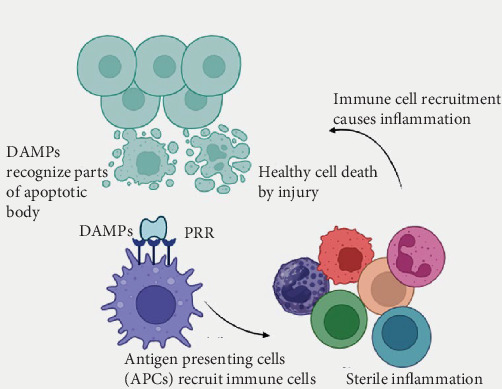
Overview of sterile inflammation.

In case of SI, it involves the recruitment of macrophages and neutrophils, the production of ROS and proteases, and the release of pro-inflammatory cytokines and chemokines. This inflammation arises when normal cells become altered or mislocalized, leading to the release of DAMPs [16, 17]. Normally, DAMPs are endogenous factors that are located inside cells and, therefore, are beyond the reach of the immune system. Under certain conditions, such as cell injury or stress, the cells are induced to release DAMPs into the extracellular environment where they bind their receptors to induce inflammation even in the absence of infection. (Figure 1) Several DAMPs are markers of SI that are pathologically linked with specific conditions [18]. The markers are termed SI marker. Some of the significant SI markers are summarized in Table 1.

Pathogenic invaders stimulate a cascade of immune responses in the host by interacting with the host's immune surveillance mechanisms through the interactions of their virulence factors. Hosts recognize conserved molecular structures known as PAMPs with the help of PRRs expressed on innate immune cells.

**Figure 2:**
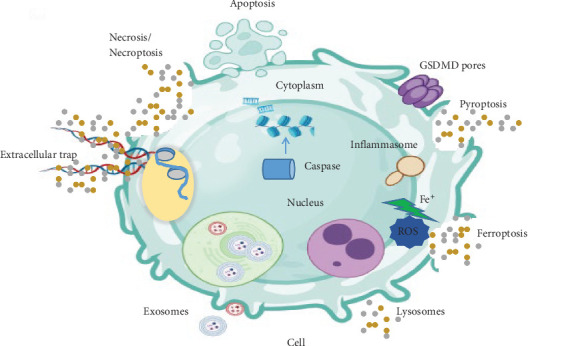
DAMP associated with different modes of cell death.

For example, TLR4 recognizes LPS, TLR3, RIG-I, and dsRNA. Binding of PAMPs to TLRs or PRRs on the cell surface activates downstream signaling pathways that lead to pro-inflammatory and antimicrobial responses mediated by intracellular factors including NF-κB, AP-1, and IRFs [21–23]. On the other hand, DAMPs activate immune responses to trauma, ischemia, cancer, and tissue damage and stimulate signaling pathways, which include MAPKs, NF-κB, and PI3K/AKT, upon binding to their specific receptors. Inflammatory disorders are often associated with an elevated concentration of DAMPs in serum [24, 25] (Figure 2).

DAMPs activate the coagulation cascade, initiating thrombus formation through the processes of initiation, amplification, and propagation. Inflammation, driven by DAMPs, also plays an important role in thrombosis. DAMPs activate immune cells, leading to the release of pro-inflammatory cytokines and eicosanoids, further promoting thrombosis. In particular, DAMPs such as HMGB1 and others are involved in linking immune activation with coagulation. The link between inflammation and thrombosis highlights the critical role of DAMPs in the development of venous thromboembolism (VTE), a significant health concern associated with mortality, cardiovascular disability, and long-term complications, particularly in situations such as infection and injury, where DAMPs increase the risk of thromboembolic events [26].

## 3. SI and Signaling Towards Genesis of Inflammasome

### 3.1. Role of PRRs

PRRs are important innate immune system sensors able to identify unique molecular patterns present on microbes, apoptotic cells, or damaged tissue. Such receptors encompass the TLRs family and NLR family that help link innate to adaptive immunity. Activation of immediate defense mechanisms coupled with tissue repair can be activated. Aberrant PRR signaling can cause either inappropriate hyperactivation of innate immunity or persistent adaptive immune responses, leading to excessive inflammation and chronic inflammatory conditions. There is a strong association between NLR gene mutations and idiopathic inflammatory diseases as evidenced by genetic studies [27], but TLR upregulation in affected tissues suggests them to be involved pathophysiologically. NLR activates inflammasomes, which enhances the formation of IL-1β and IL-18, key mediators of inflammation. Understanding the roles of TLRs and NLRs in inflammation opens up possibilities for novel therapeutic strategies to prevent or treat inflammatory disorders.

### 3.2. Role of NLRs

**Figure 3:**
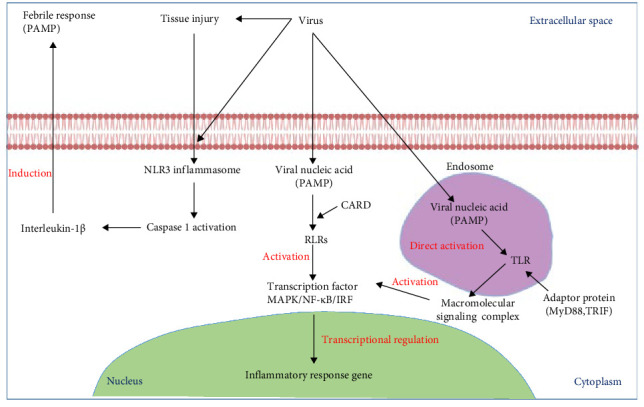
A simplified model of how viral nucleic acids (such as dsRNA, ssRNA, and dsDNA) activate PRRs. TLRs in endosomes and RIG-1-like receptors (RLRs) in the cytoplasm are directly activated by viral nucleic acids. This activation triggers the formation of macromolecular signaling complexes through adapter proteins like MyD88 and TRIF, as well as adapter motifs like CARD (caspase recruitment domains). These complexes initiate downstream signaling pathways (not depicted), leading to the activation of transcription factors (NF-kB, p38-MAP-kinase pathway, and interferon regulatory factor [IRF]), resulting in the expression of genes involved in inflammatory responses. Additionally, tissue factors released during viral injury can activate the NLRP3 inflammasome, which induces interleukin-1b febrile responses. PAMP refers to pathogen-associated molecular pattern. NLR3, NOD like receptor family, pyrin domain containing 3. CARD, Caspase recruitment domain; IRF, interferon regulatory factor; RLRs, RIG-1 like receptors; TLRs, Toll-like receptors.

NLRs can initiate sterile inflammatory processes and are mainly involved in mediating host reactions to bacterial infection [28]. Recent studies have revealed that NLRP6 inhibits NF-κB translocation and MAPK activation. Activation of NLRP6 has been linked to heightened susceptibility to both intracellular and extracellular bacteria [29]. Consequently, certain NLR family members, such as NLRP6 or NLRC5, may function as molecular switches to suppress host responses triggered by extracellular bacteria [30]. Again NLR has also played an important part in Cspase1 activation. The cytoplasm contains IL-1b in its bigger precursor form, which is broken into an active form by Caspase 1. The multimolecular complex known as the inflammasome, which forms in the cytoplasm, is responsible for Caspase 1 activation. As a part of this pathway, NLRs have the ability to trigger inflammasome activation by acting as PRR [31]. The mechanism by which viruses trigger the inflammasome is not yet known [32]. While certain viruses can activate NLRs directly, others can do so indirectly by causing membrane disruptions and tissue damage (Figure 3).

### 3.3. Role of TLRs

TLRs are single-pass receptors that play a very important role for innate immunity. Similar to PRRs, TLRs are mainly expressed by the sentinel cells viz dendritic cells or/and macrophages, as well as by nonimmune cells like intestinal epithelial cells too. TLRs consists of an extracellular domain of leucine-rich repeat motifs for ligand binding and a cytoplasmic Toll/IL-1R (TIR) domain required for intracellular signaling [33].  Table 2 lists the types of cells expressing these TLRs as well as their respective exogenous ligands [34].

**Figure 4:**
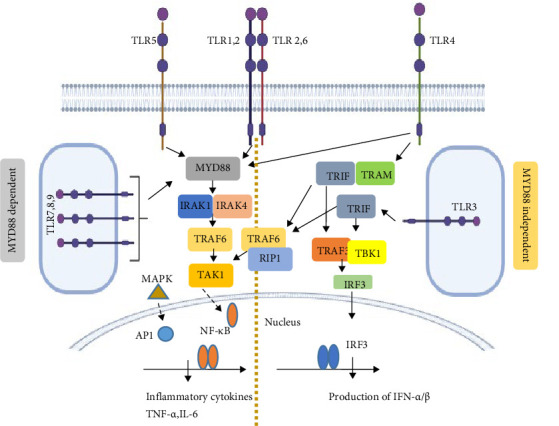
TLR signaling pathway is essential for detecting distinct microbial patterns. All TLRs, except TLR3, trigger the production of pro-inflammatory cytokines via a MyD88–IRAK–TRAF6–NF-κB dependent signaling pathway. On the other hand, the MyD88–IRAK–TRAF6 pathway is involved in the secretion of type I interferons (IFNs) also (not shown). TLR3, leads to the secretion of pro-inflammatory cytokines such as IL-1 and TNF-α through a TRIF- and RIP1-dependent mechanism. Additionally, both TLR3 and TLR4 can engage a MyD88-independent pathway, also known as the TRIF-dependent pathway, to induce the expression of Type I interferons (IFN-α, β) and IFN-inducible proteins in response to viral infections. NF-κB, nuclear factor kappa-light-chain-enhancer of activated B cells; TRAM, translocation associated membrane protein 1. AP1, activator protein 1; IL-1, interleukin-1; IRAK1, interleukin-1 receptor-associated kinase 1; IRAK4, interleukin-1 receptor associated kinase 4; IRF3, interferon regulatory factor 3; MAPK, mitogen-activated protein kinase; MyD88, myeloid differentiation primary response 88; RIP1, receptor interacting serine/threonine-protein kinase 1; TAK1, transforming growth factor beta-activated kinase 1; TBK1, TANK-binding kinase 1; TNFα, tumornecrosis factor alpha; TRAF6, TNF receptor associated factor protein family; TRIF, TIR-domain-containing adapter-inducing interferon-β.

Surface TLRs (TLR1, 2, 4, 5, 6, and 10) detect mainly microbial membrane components. However, the intracellular TLRs (TLR3, 7, 8, and 9) detect more of microbial nucleic acids (Figure 4). Nucleic acids do not generally have characteristics that are unique to microorganisms, as they are present in both pathogens and the host. Unlike bacteria, viruses lack any unique biochemistry that can be targeted by the innate immune system. Hence, the strategy of viral recognition by the immune system has been reassigned to the recognition of viral nucleic acids where TLR9 plays a very important role.The intracellular localization of TLR9 contributes to the avoidance of self-DNA recognition. An extracellular environment that promotes self-DNA degradation combined with intracellular sequestration of nucleic acid-specific TLRs establishes a carefully balanced system of nucleic acid recognition. In this way, the intracellular localization of TLR9 ensures that activation occurs only through the recognition of foreign CpG DNA and is therefore associated with an appropriate immune response and not with autoimmunity to self DNA [35].

In the context of coronary artery disease (CAD), which is driven by both lipid accumulation and inflammation, the role of TLRs, particularly TLR3 and TLR4, has gained significant attention [36, 37]. Recent studies suggest that they contribute to the pathogenesis of CAD. A study examining the expression of TLR3, TLR4, and related inflammatory mediators in monocytes, along with associated signaling proteins, found notable alterations in patients with varying degrees of coronary artery atherosclerosis [37]. Specifically, the expression of TLR3 and its downstream factor IRF-3 was significantly downregulated as the severity of coronary artery stenosis increased. In contrast, TLR4 and its signaling protein MyD88 were upregulated in patients with coronary artery stenosis, and this upregulation correlated with the number of stenosed vessels. Additionally, inflammatory cytokines such as TNF-α, IL-8, and monocyte chemo-attractant protein-1 (MCP-1) were elevated, while INF-β and IP-10 were reduced in serum as CAD severity worsened. These findings highlight the differential expression of TLR3 and TLR4 in both mononuclear cells and serum biomarkers, suggesting their involvement in the inflammatory processes of CAD. The correlation of TLR expression with the severity of coronary artery stenosis underscores their potential as biomarkers for cardiovascular risk, offering new insights into the immune mechanisms underlying CAD.

Activation of TLR causes inflammatory responses and Type I interferon (IFN) production that are important in fighting infections but can be deleterious if excessive or chronic, as noted in severe COVID-19. During the pandemic, fatal hyper-inflammation, rather than direct effects of SARS-CoV-2, was the primary cause of mortality in severe cases [38, 39]. A number of pharmaceuticals, such as chloroquine, hydroxychloroquine, darunavir, arbidol, favipiravir, lopinavir, remdesivir, ribavirin, ritonavir, IFNs, dexamethasone, and tocilizumab, has been repurposed for treatment of COVID-19. The clinical benefits of monoclonal antibodies targeting the SARS-CoV-2 spike protein have also been proven in COVID-19 treatment. However, the timing of antiviral treatment plays a crucial role in this regard [40].

Through viral infection, immune cells are activated and release several cytokines as required for the biological system. A high virus titer is associated with a cytokine storm, and such dysregulation in the body of the patient may lead to multiorgan dysfunction syndrome [41]. That is why during the COVID-19 pandemic, fatal hyper-inflammation, rather than direct effects of SARS-CoV-2, was the primary cause of mortality in severe cases [38, 39]. As a result, drugs which mainly targeted viral replication might not be effective as most of the hospitalized individuals were in the later stages of the disease.

## 4. Role of SI Molecules in Downstream Signaling Innate Immunity

Sterile inflammatory markers are molecules or substances that indicate the presence of sterile (noninfectious) inflammation, typically triggered by tissue damage or stress rather than by pathogens [18]. These markers are important in clinical settings for diagnosing and monitoring various inflammatory conditions where infection has been ruled out [42]. SI markers can be protein in nature (like C-reactive protein or serum amyloid A) produced by liver in response to inflammation or can be either pro-inflammatory cytokines (IL-1β, TNFα etc) or chemokines like IL-8 or MCP-1. Cellular markers like CD68 and DAMP can also be treated as sterile inflammatory markers. CNAs, HMGB1, vWF, and S100b proteins are mainly the example of DMAPs which are released by damaged or stressed cells, which in turn, activate innate immune response [17]. They also have a substantial influence on coagulation. The role of them in innate immunity as well as in coagulation are described in brief.

## 5. Role of CNAs

When tissue damage or vascular injury occurs, intracellular materials such as nucleic acids, histones, and other macromolecules are released. These substances, upon interacting cells of the vessel wall and circulating blood cells, can contribute to defense mechanisms like inflammation. Consequently, extracellular nucleic acids play a role in innate immunity and assist in the wound healing process [43]. Various types of extracellular RNA (eRNA) affect the integrity and barrier function of the vascular endothelium. For example, rRNA may interact with cytokines to induce hyperpermeability [44] miRNA can impact stability, and viral RNAs are recognized by TLRs [45]. Additionally, mitochondrial DNA (mtDNA) is identified as a DAMP that activates TLR9, IFN pathways, and Nod-like receptors [46]. Biswas et al. [47, 48] in their work demonstrated that how hypoxia-induced activation of eRNA can play a critical role in SI by triggering TLR3 3. This activation leads to the upregulation of cell adhesion molecules (CAMs) and enhances leukocyte infiltration, which is central to the inflammatory process in the lungs. Through the TLR3–IFN-γ–STAT1 signaling pathway, a direct link between eRNA release and the activation of inflammatory cascades have been observed, which in turn, contribute to conditions such as thrombosis, pulmonary edema, and tissue damage associated with lung injury.

A promising approach could involve the use of RNaseA or other RNase–based therapies to degrade eRNA and, consequently, block TLR3 activation. By disrupting this pathway, RNaseA treatment could reduce leukocyte recruitment and inflammation, ultimately mitigating lung injury. Additionally, strategies aimed at selectively targeting the downstream inflammatory signaling molecules, such as IFN-γ and STAT1, may offer more refined therapeutic options, potentially minimizing adverse effects associated with broad immune modulation.

CNAs and inorganic polyphosphates have also been reported to be potent activators of the coagulation cascade [49]. eRNA activates coagulation though activation of factors XII and XI in vitro and in vivo. Furthermore, it has been discovered that eRNA functions as a cofactor for factor VII-activating protease (FSAP) activation.

DNA-rich neutrophil extracellular traps (NETs) have been found to promote thrombosis. Inorganic polyphosphates, which are stored in dense bodies of mammalian platelets and secreted on platelet activation, can activate the contact pathway of coagulation and strengthen fibrin clots. Polyphosphates have been shown to accelerate factor XI activation by thrombin and factor Xa [49].

### 5.1. Role of HMGB1

**Figure 5:**
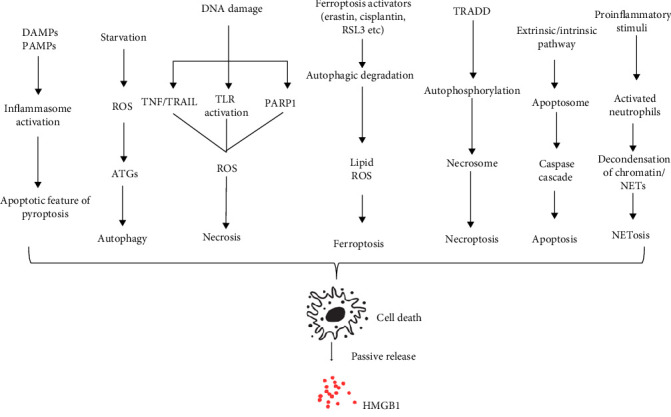
Passive release of HMGB1 during different modes of cell death.

HMGB1 is a ubiquitous nuclear protein present in the majority of eukaryotic cells. It maintains nucleosome structure and regulates gene transcription. It can also be released from cells into the extracellular space. Bioactive HMGB1 can passively diffuse from necrotic but not apoptotic cells. HMGB1 can also undergo acetylation in the nucleus and be actively released by secretory lysosomes from a variety of immune and nonimmune cells. Once secreted, HMGB1 acts as a DAMPs molecule, triggering inflammation and contributing to the pathology of various inflammatory and autoimmune diseases via activation of receptor for advanced glycation end products (RAGEs), TLR2, and TLR4 receptors. When exogenous pathogen-derived chemicals stimulate the innate immune system, HMGB1 is released actively, while invasion is absent ischemia or cell injury release HMGB1 passively. Basically this can be happened during various types of cell death, including pyroptosis, necrosis, apoptosis, necroptosis, ferroptosis, NETosis, and lysosome-mediated and autophagy-dependent cell death [50] (Figure 5).

**Figure 6:**
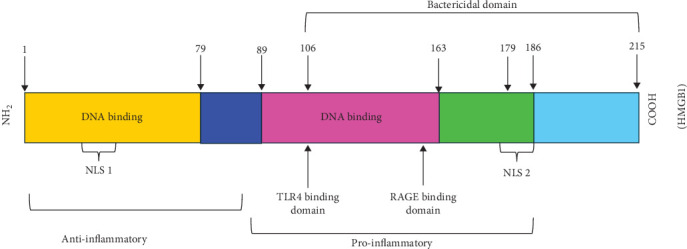
Structural organization of human HMGB1.

Established molecular mechanisms of HMGB1 binding and signaling through TLR4 reveal the signaling pathways that mediate cytokine release and tissue damage. Experimental strategies that selectively target HMGB1 and TLR4 effectively reverse and prevent activation of innate immunity and significantly reduce intensity of the damage in diverse models of sterile and infection-induced threat [51]. Many receptors bind to HMGB1 complex, but only RAGE and TLR4 are fully confirmed to act as established HMGB1 receptor [52] (Figure 6).

In 2004, Rouhiainen [53] reported that HMGB1 is expressed by platelets, even though they do not have a nucleus, and both translocated to the cell membrane and released on activation. More recently, it has been suggested that microparticles derived from activated platelets carrying HMGB1 may contribute to the chronic microvascular injury and endothelial activation seen in systemic sclerosis. Vogel et al. [54] were the first to identify that platelet-derived HMGB1 contributes to thrombosis. These investigators determined that ferric chloride-induced thrombus formation in mice mesenteric arteries was decreased in mice with a platelet-specific deletion of HMGB1, and blood from these mice had lower thrombogenicity in a collagen-coated flow chamber system. Additionally, the expression of HMGB1 on platelet surfaces was dramatically increased in trauma patients and also that the deletion of HMGB1 from platelets diminished, in a mouse model of trauma/hemorrhagic shock, platelet aggregation, microvascular thrombosis, inflammation, and organ damage. Stark and Massberg [2] have found activated platelets to be the main source of HMGB1 in venous thrombosis (VT) models in mice. Using an array of subtle experiments, they demonstrated that HMGB1 released by platelets accelerates thrombosis in inferior vena cava stenosis through trans-platelet, monocyte, and neutrophil interactions. HMGB1 is very highly modified after its translation from mRNA. Notably, Stark and Massberg [2] found that the oxidized, disulfide isoform of HMGB1 plays a critical role in the process of platelet aggregation, expression of tissue factor (TF) on monocytes, extracellular traps in neutrophils, and subsequent development of thrombosis via venous pathway [55].

### 5.2. Role of vWF

vWF is a large multimeric glycoprotein (GP) produced in endothelial cells and megakaryocytes and present in subendothelial matrix, blood plasma, and platelets. It works to support thrombus formation not only with respect to maintaining platelet adhesion to sites of injury but also platelet–platelet cohesion or aggregation. Platelets respond rapidly to alterations of endothelial cells by attaching firmly to the site of lesion where exposure of subendothelial components may have occurred. The first layer of platelets is in contact with the thrombogenic surface (adhesion), whereas subsequent growth of the hemostatic plug depends on platelet-to-platelet interactions (aggregation). Both aspects of platelet function are influenced by vWF interactions with specific platelet membrane receptors. Multiple domains of vWF are involved in securing initiation and growth of platelet plugs [56].

**Figure 7:**
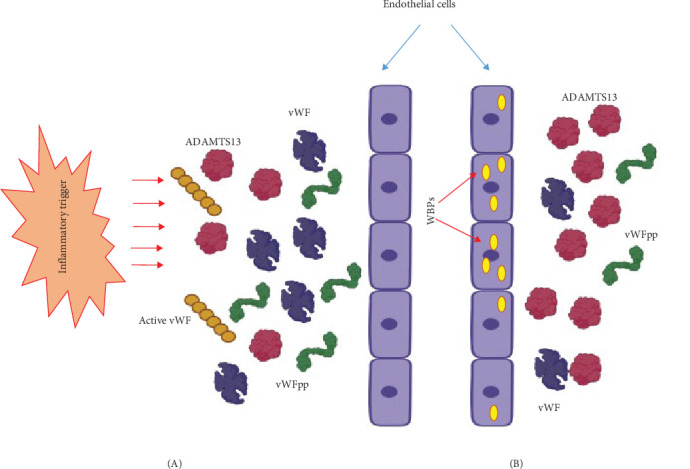
Endothelial cells become activated in response to an inflammatory stimulus, which causes vWF and its propeptide vWFpp (yellow in color) to be released in massive quantities from Weibel–Palade bodies (WPBs) (A). vWF and vWFpp levels are consequently higher than in the basal state (B), and measuring the VWFpp/VWF ratio may aid in differentiating between acute and chronic inflammation.

Notably, elevated vWF levels are associated with inflammation and diseases like arteritis, diabetes, and sepsis. vWF also orchestrates the synthesis of Weibel–Palade bodies (WPBs), containing inflammatory players like P-selectin, IL-8, and eotaxin-3. Endothelial cells, positioned at the blood–tissue interface, swiftly respond to inflammatory stimuli by altering their phenotype to support inflammation. This response includes the rapid release of WPBs, predominantly loaded with vWF, into the extracellular milieu, thus, making vWF an acute-phase protein (Figure 7A,B).

### 5.3. Role of S100b Protein

**Figure 8:**
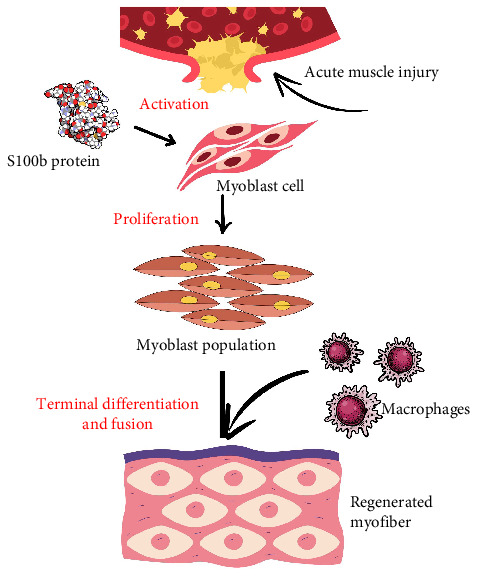
Regeneration of skeletal muscles by appropriate levels of S100b protein after acute muscle injury.

S100b is a member of the cytosolic calcium binding protein family [57], which includes calcium-binding proteins involved in various physiological processes such as inflammation, apoptosis, calcium regulation, migration, and energy metabolism [58]. These proteins play essential roles both intracellularly and extracellularly, influencing cellular responses to injury and stress. For instance, after acute muscle injury, S100b facilitates skeletal muscle regeneration by promoting myoblast expansion and attracting macrophages, thereby enhancing tissue repair and collagen deposition through interactions with FGFR1 and the RAGE receptor (Figure 8).

In pathological conditions like breast cancer, diabetes, and tissue injuries in the brain and heart, S100b expression is often elevated [52, 57–59]. It has also been explored as a biomarker to differentiate between hemorrhagic and ischemic stroke, with increased levels observed in hemorrhagic stroke. Interestingly, S100b levels in clots were not significantly affected by thrombolytic treatment, highlighting its potential role in coagulation during stroke as shown by Rossi et al. [60] in their work. S100b's association with innate immune cells, such as macrophages and neutrophils, underscores its involvement in the inflammatory response. As a DAMP molecule, S100b is released from damaged cells and acts as an endogenous danger signal to trigger inflammation. It regulates macrophage-mediated inflammation by upregulating pro-inflammatory cytokine production, which can exacerbate inflammation in stroke. Additionally, S100b induces neutrophil migration to injury sites, where neutrophils contribute to ischemic damage through neuronal death, blood–brain barrier disruption, and brain edema. NETs can further activate thrombotic processes, emphasizing the dual role of S100b in both innate immunity and coagulation during stroke [60].

### 5.4. Role of SI in Thrombosis

SI plays an important role in the pathogenesis of thrombosis by initiating complex interactions between the immune and coagulation systems. In the context of atherosclerosis, cardiovascular diseases, and COVID-19, SI-mediated thrombosis, or immunothrombosis, contributes significantly to pathological clot formation and endothelial dysfunction. Immunothrombosis is a reciprocal interaction where the innate immune system and coagulation cascade converge, often amplifying thrombotic risk in inflammatory settings. This process involves a series of interconnected mechanisms, including immune complex deposition, complement activation, platelet aggregation, and TF expression, which drive thrombus formation. One critical mechanism in SI-driven thrombosis is the deposition of antigen–antibody complexes in the vessel walls, a hallmark of Type III hypersensitivity reactions. When immune complexes accumulate in blood vessel walls, they induce endothelial damage, leading to the exposure of the subendothelial matrix. This exposure promotes platelet adhesion and aggregation, a precursor to thrombus formation. As a result, SI-induced endothelial injury amplifies the procoagulant environment, fostering the formation of blood clots [61–64].

Additionally, the activation of the complement system plays a significant role in this process. Complement components, particularly C3a and C5a, act as potent inflammatory mediators, recruiting and activating neutrophils and monocytes at the site of immune complex deposition. These activated immune cells release further inflammatory cytokines and mediators, which contribute to the activation and aggregation of platelets, escalating thrombotic risk [65].

Neutrophils also contribute to thrombosis through the release of NETs. NETs act as scaffolds for platelet adhesion and fibrin deposition, thereby enhancing clot formation. In the context of SI, NETosis (the release of NETs) serves as a crucial mechanism that links inflammation to coagulation by facilitating the retention of TF and extracellular vesicles, both of which are essential for thrombus formation [66]. Furthermore, the upregulation of TF expression in endothelial cells and monocytes in response to immune complex deposition contributes to thrombin generation, ultimately resulting in fibrin clot formation [67].

Moreover, platelets express Fcγ receptors (FcγRIIA), which bind to immune complexes, triggering platelet activation and degranulation [68]. FcγRIIA-mediated platelet activation significantly amplifies platelet aggregation and clot formation in SI-associated thrombosis, platelet activation, along with TF expression, creates a positive feedback loop that enhances thrombotic tendencies, especially in patients with underlying inflammatory conditions, including cardiovascular disease and COVID-19.

**Figure 9:**
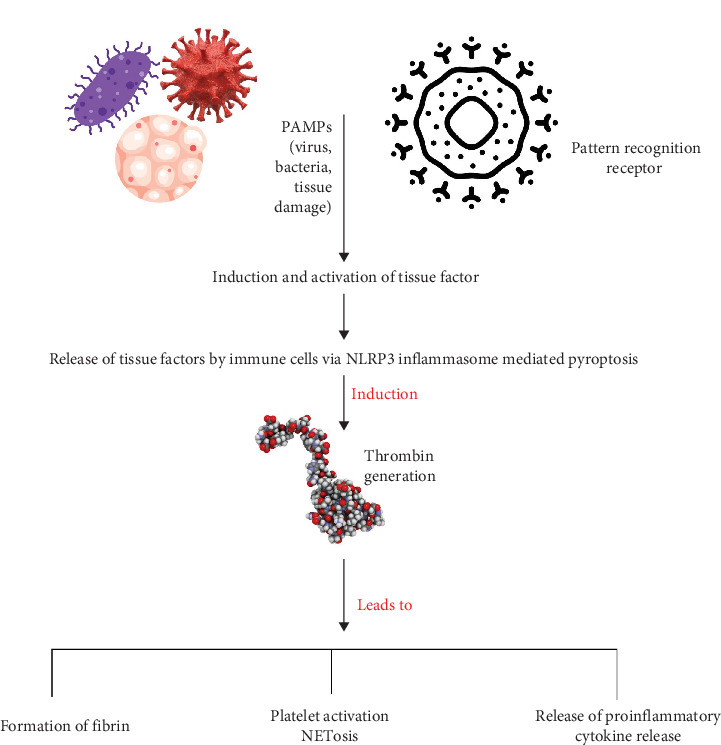
Molecular mechanism implied in immunothrombosis/thromboinflammation. Upon activation of immune cells, tissue factor (TF) is decrypted and released from these cells. This activation also triggers pyroptosis via the NLRP3 inflammasome. Consequently, thrombin is generated, which leads to platelet activation and NETosis. These events enhance fibrin formation and increase the release of proinflammatory cytokines.

In pathological conditions like sepsis, myocardial infarction, stroke, and COVID-19-associated coagulopathy, the interplay between inflammation and coagulation cascades can lead to severe complications. PAMPs from bacterial and viral infections activate PRRs such as TLRs, leading to the induction of TF. This process, referred to as TF decryption, significantly amplifies its procoagulant activity, driving the immunothrombosis process. In response to inflammation, TF interacts with activated factor VII, triggering the extrinsic coagulation pathway and generating thrombin, which further activates platelets and promotes fibrin clot formation (Figure 9).

In COVID-19, SI exacerbates thrombotic events, with viral components such as SARS-CoV-2 inducing widespread immune activation and enhancing endothelial dysfunction. The elevation in inflammatory cytokines, alongside the release of TF and immune complexes, exacerbates coagulation abnormalities and increases the risk of VTE and other thrombotic complications.

SI-induced thrombosis represents a critical intersection of immune activation and coagulation dysregulation. The deposition of immune complexes, complement activation, NET release, and the upregulation of TF are key processes contributing to thrombus formation in both cardiovascular diseases and COVID-19. Understanding the molecular mechanisms of immunothrombosis highlights the complex relationship between inflammation and coagulation, providing valuable insights into potential therapeutic targets aimed at reducing thrombosis in these inflammatory conditions.

## 6. Relation Between Gut Microbiota-Derived Pattern Recognition and SI: Involvement in Thrombosis

Studies on microbiomes have revealed clear variations in microbial metabolites and gut microbiome profiles between healthy individuals and patients suffering from venous or arterial thrombosis. The gut microbiota has emerged as an environmental risk factor that influences thrombotic phenotypes in a number of cardiovascular disorders. Metabolites produced from the gut microbiota have been demonstrated to act on vascular cell types and induce thrombus formation, in addition to impaired gut barrier function. Consequently, meta-organismal pathways that connect the host immune system and the metabolic abilities of gut microbes have become promising targets for new drugs and diagnostic markers [69]. A recent review by Khuu et al. [69] gave a fascinating correlation between gut microbiota derived pattern recognition and SI in thrombosis. The gut microbiota is involved in the regulation of gut epithelium permeability through PAMPs. When PAMPs are recognized by TLRs or nucleotide-NLRs, the intestinal epithelial barrier weakens, allowing bacterial components to translocate into the bloodstream. From there, these PAMPs move along the gut–liver axis and enter the liver microcirculation, where they cause activation of TLR2 on hepatic endothelial cells. This activation will result in increased expression and the release of vWF into the blood. It binds to the extracellular matrix (ECM) in the vascular wall and GP IIb/IIIa on platelets, which prompts platelet activation as well as their adhesion to endothelial cells promoting thrombus formation. Besides this, TLR2 or TLR4 signaling in platelets activates them and the platelets activated by LPS-induced TLR4 signaling release α-granules containing thrombospondin 1 (TSP1), which further activates other platelets through binding to platelet GP 4 (CD36). Circulating LPS also initiates innate immune responses wherein activated monocytes secrete TF, the main initiator of the coagulation cascade and activated neutrophils extrude NETs. The endothelium is also activated by TLR2 or TLR4 signaling, changing its inflammatory and adhesive properties. This activation results in the secretion of prothrombotic vWF, coagulation factor VIII, pro-inflammatory mediators such as IL-6 and IL-8, and the surface expression of intercellular adhesion molecule 1 (ICAM1), which facilitates leukocyte–endothelial cell interactions [69].

### 6.1. Inflammation and Thrombosis

**Figure 10:**
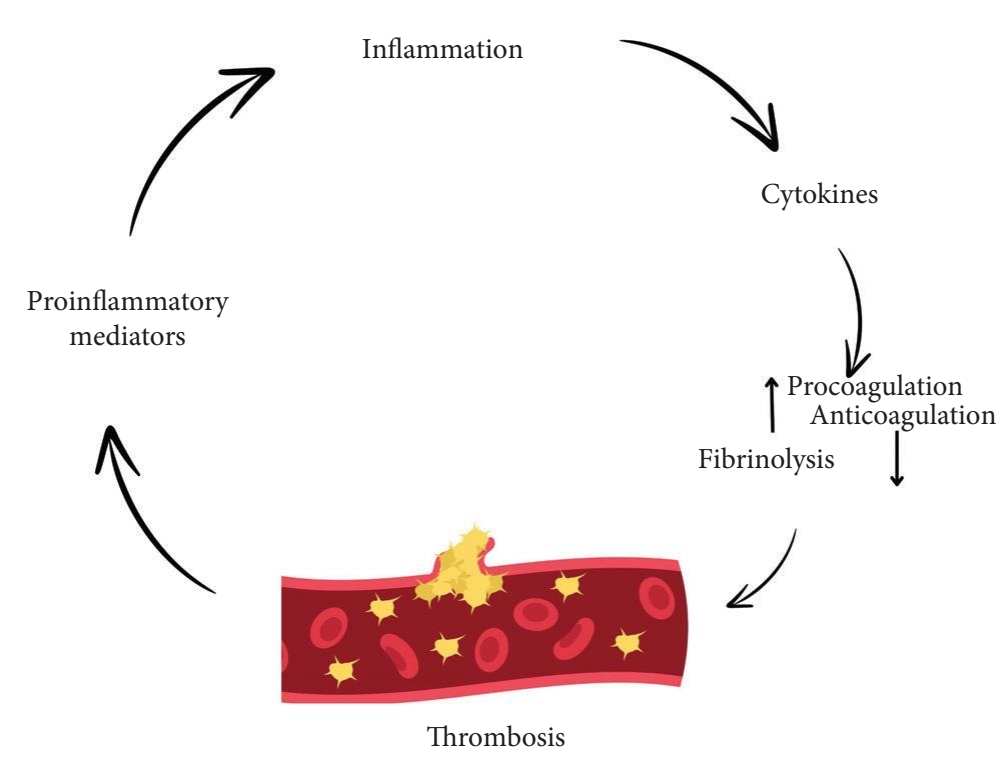
Thrombosis and inflammation: The processes of hemostasis and thrombosis are closely interconnected. Key cytokines such as TNFα, IL-1, and IL-6 can contribute to a prothrombotic environment.

Inflammation and thrombosis are interconnected. There has been numerous data and indications where close relation between inflammatory immune response and haemostasis have been shown. Factors which influence the formation of blood clots of VTE are not only limited to the coagulation system alone rather there are reports which suggests the direct involvement of immune system in the formation of thrombosis. In blood, a delicate balanced exists between procoagulant and anticoagulant factors. Inflammation can disrupt this balance and leads to a pro-thrombotic state, thereby increasing the risk of thrombosis. TNFɑ, IL-1,IL-6, C-reactive protein are such key inflammatory markers which involved in this process [70, 71] (Figure 10). Inflammatory mechanisms can upregulate procoagulant factors, leads to platelet activation, inhibit fibrinolytic activity, functional impairment of natural anticoagulants, activation of TF mediated coagulation, et cetera.

Some studies suggest an evolutionary link between inflammatory mechanisms and the coagulation/anticoagulation processes [72]. Structural observations, such as the homology between TF and cytokine receptors, illustrate this interaction between inflammation and thrombosis. Inflammation and thrombosis are interrelated in a cyclical manner, where each can trigger the other. It is now well acknowledged that coagulation regulators, which were formerly thought to just assist in the haemostasis apparatus and facilitate blood clotting, also control cellular functions, such as inflammation. The main participants in thrombosis are activated platelets, which can either directly or indirectly activate immune cells to cause inflammation and immunological responses. Regarding this, the pathophysiology of cardiovascular disease is significantly influenced by neutrophil activation and NET formation, which establish a connection between thrombosis and inflammation, a phenomenon known as thrombo-inflammation. Inflammatory health issues, like diabetes, can increase the risk of developing blood clots. In other words, conditions characterized by chronic inflammation can make it more likely for a person to experience thrombosis, which is the formation of blood clots in blood vessels. This highlights the link between inflammation and the increased likelihood of clot formation, suggesting that managing inflammation might be important in preventing thrombotic events [73, 74]. Changes in the regulation of coagulation proteases and platelet activation coincide with the activation of the inflammatory response. Therefore, antithrombotic therapies which have a dual mode of action, and not only prevent clotting but can confer cytoprotective effects are beneficial in resolving thrombo-inflammation. Identifying the potential therapeutic targets of immunothrombosis could be a great promise of covering therapeutic gaps in cardiovascular medicine with anti-inflammatory approaches and might also help to fight thrombotic complications in infectious diseases, including COVID-19 [2, 75]. Thrombin, a procoagulant, cleaves fibrinogen into fibrin, forming a fibrin mesh that creates an initial platelet plug. Thrombin also has inflammatory effects, such as activating the transcription factor NF-κB and pro-inflammatory cytokines like TNF-α and IL-1, which further promote both inflammation and thrombosis [76]. Another key molecule, P-selectin, links inflammation and thrombosis by facilitating the adhesion of leukocytes and platelets. The release of P-selectin from inflamed tissues increases vein wall cell infiltration, contributing to thrombosis [77]. Under normal conditions, TF is present in very low amounts. Inflammation increases TF expression, shifting the hemostatic balance towards thrombosis. Elevated TF levels enhance inflammatory factors such as TNF-α and IL-1α, which further promote coagulation [78]. Additionally, optimal procoagulant lipid surface expression requires the combination of collagen, thrombin, other agonist cell molecules, and the complement C5b9 complex [79]. For effective immunity against infections, the body must recognize the presence of microorganisms through molecular patterns, either by PAMPs or DAMPs.

### 6.2. Role of Hypoxia in Induction of Inflammation

**Figure 11:**
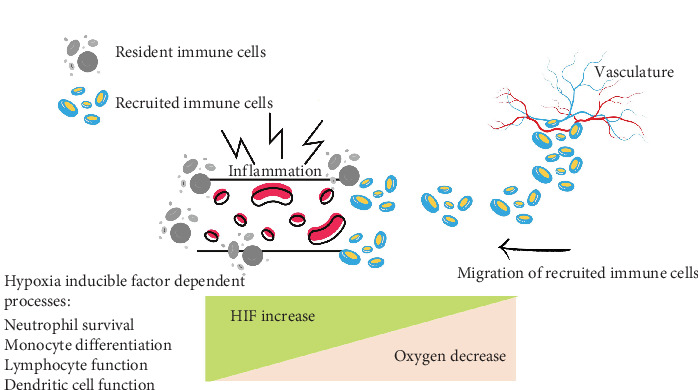
Hypoxia is crucial in the inflammatory process. In chronic inflammation, the oxygen demand in affected tissues increases while the oxygen supply decreases, resulting in tissue hypoxia. This oxygen shortage leads to heightened activity of hypoxia-inducible factors (HIF) in both resident and infiltrating immune cells. The elevated HIF activity then alters the function of these immune cells, affecting the overall inflammatory response and its severity.

Hypoxia and inflammation are interrelated. At high altitudes, elevated levels of inflammatory mediators are observed, indicating that hypoxia induces inflammation [80]. Conversely, patients with inflammatory bowel disease exhibit increased levels of hypoxia-inducible factors (HIFs), suggesting that inflamed tissues often become hypoxic [81]. This interdependent relationship can be termed “inflammatory hypoxia.” This condition arises from the increased oxygen demand by infiltrating inflammatory cells, such as neutrophils, coupled with reduced oxygen supply to the inflamed tissues. Neutrophils at the inflammation site require substantial oxygen to produce reactive oxygen species (ROS) for pathogen destruction, leading to hypoxia in the inflamed area. Conversely, hypoxic conditions within cells lead to higher HIF levels. Erythropoietin (Epo) is a hypoxia-responsive gene that is expressed under low-oxygen conditions, resulting in HIF accumulation, which then promotes the expression of genes involved in hypoxic adaptation, including angiogenesis. Thus, HIF is regarded as a ‘master regulator' of the cellular response to hypoxia [82] (Figure 11).

Another example of hypoxia-induced inflammation is observed in individuals with acute mountain sickness (AMS), who show high levels of IL-6, IL-6 receptor, and C-reactive protein—markers of inflammation. In contrast, in inflammatory bowel disease, the entire mucosa becomes highly hypoxic [83].

### 6.3. Role of Hypoxia in Thrombogenesis

**Figure 12:**
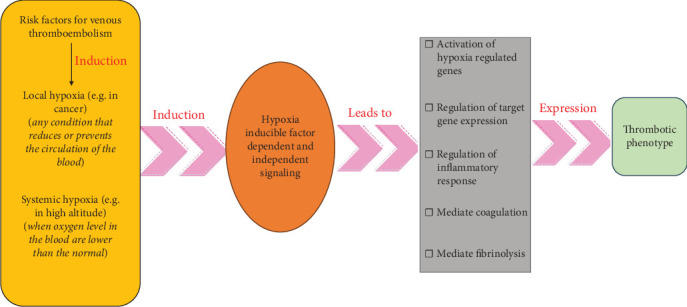
Hypoxia links thrombosis risk factors to blood clot formation. When these risk factors induce hypoxia, it activates both HIF-dependent and independent signaling pathways, which then trigger the expression of hypoxia-regulated genes involved in coagulation and fibrinolysis. Targeting these hypoxia-regulated genes could offer a promising strategy for preventing thrombosis.

Imbalances between oxygen supply and usage can lead to hypoxia. Numerous studies have shown that thrombus formation is heightened under hypoxic conditions [84]. Hypoxia is regulated by HIFs, which promote the expression of factors involved in thrombus formation. Thrombus formation can be modulated through HIF-dependent or HIF-independent pathways. In the HIF-dependent pathway, hypoxic cells accumulate HIF1 and HIF2 in the nucleus, forming an active complex that regulates coagulation factors such as prothrombotic TF and plasminogen activator inhibitor (PAI). Further research is needed to elucidate the roles of HIFs in different cell types with regard to thrombus formation [85]. Additionally, HIF-dependent mechanisms include the modulation of integrin receptors. Hypoxia enhances the expression and function of endothelial integrin receptors, leading to increased expression of hypoxia-inducible integrins and subsequent thrombus formation [86]. Another mechanism involves HIF activation and the formation of NETs, which are meshes of DNA fibers, histones, and antimicrobial proteins. Under inflammatory and hypoxic conditions, HIF1 and NET activation increase endothelial activation, contributing to thrombosis [87] (Figure 12).

## 7. Thrombotic Disorder in Coronavirus Disease 2019 (Covid19): Role of SI

### 7.1. Overview of Thrombotic Disorders and COVID-19

Patients with COVID-19 show a diverse level of severity that ranges from asymptomatic to acute phase of multiorgan dysfunctions. Although most of the COVID-19 patients have respiratory infection, some of the patients develop coagulation abnormalities such as thrombocytopenia, disseminated intravascular coagulation (DIC), and VT [87]. Recent studies showed increased incidence of deep vein thrombosis (DVT), myocardial infraction, and ischemic stroke in patients with Covid19 [88]. Coagulation activation as well as widespread vascular inflammation has been shown to be present in COVID-19 patients with severe outcome.

### 7.2. Coagulation Abnormalities and Inflammation in Severe COVID-19

Emerging reports show that a subset of COVID-19 infected patients, who develop severe disease, are commonly shown with prolonged activated partial thromboplastin time (APTT), delayed prothrombin time (PT), higher d-dimer and fibrin degradation products (FDPs) levels, increased thrombin–antithrombin (TAT) complex, and decreased antithrombin (AT) in plasma samples [89]. There was a report of cohort study conducted in Germany with 100 patients who recovered from COVID-19, revealed that 78% of the patients presented with abnormal cardiovascular magnetic resonance (CMR) imaging (MRI), and 71% presented elevated levels of high sensitivity troponin T at the time of the CMR [90].

### 7.3. Cardiovascular Manifestations and the Role of Angiotensin-Converting Enzyme 2 (ACE2)

**Figure 13:**
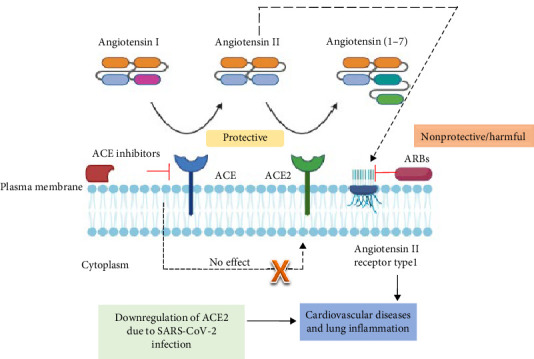
Angiotensin II, the main effector molecule in the RAAS, is upregulated in many pathological conditions, for which inhibition of angiotensin II by RAAS inhibitors is a common therapeutic approach. Angiotensin-converting enzyme (ACE) generates angiotensin II from angiotensin I, while ACE2 counteracts this by converting angiotensin II into angiotensin (1–7). Consequently, ACE2 plays a protective role against cardiovascular disease and lung injury. In the context of COVID-19, the downregulation of ACE2 due to SARS-CoV-2 infection may contribute to cardiovascular damage. ARB, angiotensin II receptor blocker.

The potential correlation between COVID-19 and cardiovascular problems may arise from the way SARS-CoV-2 interacts with one or more critical pathways that regulate the cardiovascular system [91]. Recent research has demonstrated that the renin–angiotensin–aldosterone system (RAAS) in COVID-19 is essential for the virus's ability to bind to ACE2 on the surface of epithelial cells, which allows the virus to be recognized [92]. Interestingly ACE2 is not only expressed in nasopharyngeal and lung cells but also in blood vessels, heart, kidney, testicles, and brain. In fact, ACE2 was first cloned from human heart and found to be highly expressed throughout the endothelia of coronary and renal blood vessels [93], which may suggest the importance of ACE2 in cardiovascular system. Indeed, a major role of ACE2 among others is to inactivate angiotensin II by proteolytically converting it to angiotensin 1–7 [94]. This, thus, places ACE2 in a critical position as a negative regulator of the RAAS. Many reports indicate that SARS-CoV2 entry is associated with downregulating ACE2 activity [95], suggesting that RAAS may be augmented in COVID-19 patients. Drugs used to reduce cardiovascular risk such as ACE2 inhibitors and angiotensin II receptor blocker (ARB) have multiple effects that might influence susceptibility to the sensitivity of Covid19 [96] (Figure 13). The higher affinity of SARS-CoV2 to human ACE2 compared to other corona viruses [97] and the downregulation of ACE2 in COVID-19 may explain the peculiar cardiovascular manifestations seen in vulnerable patients.

## 8. Impact of Viral dsRNA and TLR3 Activation on Hypertension and Thrombosis

The majority of viruses produce dsRNA during the replication stage. This RNA can function as a DAMP or PAMP, which can activate multiple antiviral pathways that are essential for preventing early viral invasion, such as TLR3 [98], Type I IFN generation, RNA degradation, and the activation of dsRNA-dependent protein kinase (PKR) are all components of TLR3 downstream signaling. Increased PKR activity through dsRNA sensing has been linked to the pathophysiology of vascular disorders by inducing oxidative stress, apoptosis, and inflammation. Although several viral infection including human herpes-virus and cytomegalovirus and human immunodeficiency virus 1 are associated with hypertension and increased blood pressure, the mechanism behind viral infection and their contribution to hypertension is not fully understood. dsRNA might act as missing link since TLR3 recognizes dsRNA produced by most viruses at the replication stage. As evidence, nasal instillation of Poly I:C, a synthetic dsRNA that activates TLR3, led to the development of the ARDS and a cytokine storm [99] both of which are often observed in severe COVID-19 patients. Interestingly mice lacking in TLR3 (Tlr3^−/−^) did not develop elevated blood pressure upon angiotensin II infusion, indicating that TLR3-TRIF activation is a prerequisite for hypertension [100]. Another study demonstrated that SARS-CoV-2 infection of primary nasal epithelial cells influenced iPSC-derived alveolar type 2 (iAT2) cells and cardiomyocytes (iCMs), which model host tissues likely affected in clinical infection. In this context, iAT2 and iCM did not undergo direct infection but instead exhibited perinuclear localization of viral dsRNA and PKR activation in response to inflammatory signals from the infected epithelial cells [101]. As COVID-19 infection becomes increasingly associated with systemic and multiorgan involvement, including cytokine release syndrome and thromboembolic, vascular, and cardiac events, it could be a consequence of TLR3 activation. TLR3 activation via dsRNA elevates blood pressure, a characteristic shared by various pathological conditions mentioned earlier. Coronaviruses, adept at evading host antiviral pathways induced by viral dsRNA, appear to overactivate the TLR3-related pathway, potentially exacerbating damage to patients and promoting the development of cardiovascular complications, including hypertension. TLR3 activation is known to contribute to the development of conditions such as preeclampsia [102] and to induce endothelial dysfunction/damage and oxidative stress. The influx of SARS-CoV-2 into cells may lead to an exaggerated activation of the TLR3-related pathway, further compromising patients and increasing the risk of various cardiovascular complications.

### 8.1. Endothelial Dysfunction and Thrombotic Risk in COVID-19

Endothelial injury leads to the loss of protective molecules and the expression of adhesive molecules, procoagulant activities, and autogenic factors, resulting in the development of thrombosis, smooth muscle cell migration, proliferation, and atherosclerosis. Conversely, endothelial dysfunction contributes to the pathophysiology of COVID-19. This suggests a potential role for vWF, which acts as a marker of SI, in COVID-19 associated coagulopathy. Beyond its presence in plasma, vWF is deposited in sub-endothelial spaces where it binds to Type VI collagen. Following endothelial damage, subendothelial vWF is released, undergoes further multimerization through disulfide bonds, and becomes activated, exposing domains that bind to platelets and collagen. Consequently, active vWF multimers serve as molecular adhesives, promoting platelet aggregation and thrombosis by binding platelets to sub-endothelial collagens. In a single-center cross-sectional study as reported by Ali and Spinler [103], levels of vWF antigen and activity were found to be three times higher in non-intensive care unit (ICU) COVID-19 patients compared to a control group. In ICU COVID-19 patients, vWF concentration and activity were further elevated compared to non-ICU patients. This observation (elevation of vWF levels in non-ICU patients) presents an interesting aspect of endothelial dysfunction and its possible role in thrombotic risk. vWF is a GP that plays a critical role in platelet adhesion and aggregation, particularly at sites of vascular injury. It is released into circulation by endothelial cells, and its elevated levels often signal endothelial activation or injury, which are early indicators of endothelial dysfunction.

In the context of non-ICU patients, elevated vWF levels might reflect a less severe or early manifestation of endothelial dysfunction, which could be triggered by various conditions such as chronic inflammation, comorbidities (e.g., hypertension and diabetes), or vascular risk factors. This early dysfunction could precede more significant complications such as thrombosis. While ICU patients tend to show more pronounced endothelial dysfunction due to acute illness and hemodynamic instability, non-ICU patients with elevated vWF may still be at risk of thrombotic events, albeit to a lesser extent or over a longer timeline.

The clinical implications of these findings suggest that non-ICU patients with elevated vWF may be at a higher risk for developing thromboembolic events, such as DVT, pulmonary embolism (PE), or even stroke, especially in the presence of additional risk factors like immobility or hypercoagulability. However, the precise mechanisms linking vWF elevation to thrombotic events in this population require further exploration. It would be beneficial to examine whether elevated vWF could serve as an early biomarker for identifying patients at higher risk of thrombosis and whether targeted interventions, such as anticoagulation or anti-inflammatory treatments, could mitigate this risk.

### 8.2. Potential Therapeutic Strategies Targeting SI in Thrombosis and Cardiovascular Diseases

The pathophysiology of cardiovascular disease is influenced by several biological mechanisms. All of them have generally been referred to as “inflammatory;” however, this oversimplification does not accurately convey the heterogeneity of the processes involved. There is multiple experimental evidence which suggest that the specific targeting of multiple biological processes can result in reducing the injury and reparative processes can be enhanced by precisely targeting certain biological systems [104]. Targeting SI can be a probable therapeutic strategies in in thrombosis and cardiovascular diseases in this regard.

Since HMGB1's circulating levels rise soon after ischemia/reperfusion and its inhibition prevent ischemic injury, it plays a pathogenic role in ischemic injury. Following myocardial damage, RAGE, TLR2, and TLR4 expression levels rise allowing HMGB1 to efficiently mediate an inflammatory response and so promoting the advancement of ischemic injury. Notably, several HMGB1-inhibiting agents like ethyl pyruvate, green tea, and adrenomedulin can preserve cardiac function after myocardial ischemic or septic insults which suggest that it can be a potential therapeutic strategies in thrombosis and cardiovascular diseases [105].

A study by Rossi et al. [60] has also observed the role of S100b as a potential biomarker in acute ischemic stroke (AIS) clots for postthrombectomy intracranial hemorrhages. Evidence indicates that elevated levels of S100b in the blood of AIS patients correlate with a higher rate of intracranial hemorrhage following thrombolytic therapy. Additionally, colocalization studies revealed that S100b in retrieved AIS clots was associated with macrophages, neutrophils, and some T-lymphocytes, implying that it may influence thrombo-inflammatory activity [60]. Again, there are reports that inflammation promotes thrombosis via a vWF-mediated mechanism. vWF plays an essential role in maintaining the balance between blood coagulation and bleeding, and inflammation can lead to aberrant regulation [106]. Based on these above observations, we can say that the elucidation of the mechanism of inflammation-mediated thrombosis may identify new treatment options, that effectively prevent harmful clotting while still allowing the body to form necessary clots to stop bleeding when needed.

## 9. Conclusion

In conclusion, SI plays a critical role in the pathophysiology of COVID-19, contributing to tissue damage, thrombosis, and immune responses. The activation of the innate immune system in the absence of direct pathogen involvement triggers the release of DAMPs, which activate PRRs like TLRs and NLRs, leading to the formation of inflammasomes and subsequent immune activation. This process generates pro-inflammatory cytokines and ROS, exacerbating tissue injury, systemic inflammation, and a prothrombotic state. The inflammatory response in COVID-19 enhances endothelial dysfunction and hypercoagulability, which are key drivers of thrombosis, as evidenced by elevated markers such as vWF and CNAs. Understanding the link between SI and thrombosis opens the door to targeted therapeutic approaches aimed at mitigating thrombotic complications in COVID-19 patients. The exploration of immunomodulatory and anticoagulant therapies holds promise for addressing the inflammatory and thrombotic abnormalities in COVID-19 [107]. Drugs like tocilizumab and eculizumab, which target key inflammatory pathways, have shown potential in improving outcomes by reducing cytokine release and complement-mediated thrombotic microangiopathy [108]. Other strategies, including statins, ACE inhibitors, and low-molecular weight heparin (LMWH), aim to reduce endothelial injury and prevent thrombosis. Furthermore, the use of Janus kinase (JAK) inhibitors is under investigation for their ability to modulate inflammation and inhibit viral entry. However, the risk of VTE with certain agents necessitates careful monitoring and further clinical trials [109]. Future research should focus on translating the mechanistic insights of SI into clinical interventions for thrombosis. Randomized clinical trials targeting HMGB1 or TLR4 inhibition could clarify their therapeutic potential in high-risk cardiovascular patients. Similarly, IL-1β antagonists or NLRP3 inflammasome inhibitors may be tested in COVID-19-associated coagulopathy to evaluate their ability to reduce thrombo-inflammatory complications. Repurposing agents with dual anti-inflammatory and antithrombotic effects, such as colchicine or statins, represents another promising avenue. Importantly, biomarker-driven studies incorporating SI markers (e.g., vWF, HMGB1, and eRNA) into patient stratification may enable precision medicine approaches for preventing and treating thrombotic events.

## Figures and Tables

**Table 1:** Receptors for disease associated molecular pattern (DAMP) involved in sterile inflammation and diseases [19, 20].

Origin	Localization	Expression pattern	Major DAMPs	Receptors	Pro-inflammatory functions	Major inflammatory diseases
Extracellular matrix	—	Ubiquitous, high in dendritic cells monocytes, macrophages neutrophils and endothelial cells	Biglycan	TLR2, TLR4, NLRP3	Promotes the production of pro-inflammatory cytokines and chemokines	IRI, RA, cancer, and other inflammatory disease
			Decorin	TLR2, TLR4		
			Versican	TLR2, TLR6, CD14		
		Ubiquitous, high in dendritic cells monocytes, macrophages, and neutrophils	Heparan sulfate	TLR4	Promotes the production of pro-inflammatory cytokines, chemokines, and IFN-1	IRI, RA, cancer, and other inflammatory disease
			Fibronectin (EDA domain)	TLR4		
			Fibrinogen	TLR4		
			Tenascin C	TLR4		

Intracellular compartments	Cytosol	—	Uric acid	NLRP3, P2X7	Activate the NLRP3 inflammasome, induce the release of IL-1β in gout activates the NLRP3 inflammasome in macrophages, and promotes inflammation	Gout, renal disorder, insulin resistance, and Type 2 diabetes
		Ubiquitous, high in dendritic cells, monocytes, macrophages neutrophils, and endothelial cells	S100 proteins	TLR2, TLR4, RAGE	Promotes the expression of pro-inflammatory genes, as well as cell migration, proliferation, and apoptosis	Diabetic vascular complications, CVD, AD, cancer and other inflammatory diseases
		Ubiquitous, high in dendritic cells, monocytes, macrophages,NK cells, and neutrophils myeloid cells, epithelial cells, endothelial cells, and fibroblasts	HSPs including HSP60 and HSP70	TLR2, TLR4, TREM1, TREM2	Promotes the production of pro-inflammatory cytokines chemokines, IFN1	IRI, RA, cancer and other inflammatory diseases, Myocardial infarction, atherosclerosis, ureteral obstruction, AD, NHD, and other neurodegenerative diseases
		Ubiquitous, high in epithelial cells, neutrophils, dendritic cells, monocytes and macrophages	ATP	P2X7, P2Y2	Promotes migration and activation of various immune cells	Chronic lung disease, asthma, hepatitis, atherosclerosis
		Mainly in dendritic cells	F-actin	DNGR-1	Promotes dendritic cell antigen cross presentation, inhibits IL-10 production	Cancer, atherosclerosis
		Dendritic cells, neutrophils, monocytes, and macrophages	Aβ	TLR2, NLRP1, NLRP3, CD36, RAGE	Promotes IL-1β and IL-18 secretion and initiates pyroptosis	T2D, NASH, gout, atherosclerosis, AD, IRI
	Nuclear	Ubiquitous, high in dendritic cells, monocytes, macrophages, and neutrophils	Histones	TLR2	Promotes the production of pro-inflammatory cytokines and chemokines	IRI, RA, cancer, and other inflammatory diseases
		Ubiquitous, high in DCs, monocytes, macrophages, and neutrophils	HMGB1	TLR2, TLR4, TLR9	—	—
		Ubiquitous, high in dendritic cells, monocytes, macrophages neutrophils, and endothelial cells	HMGN1	TLR4	Promotes the production of pro-inflammatory cytokines, chemokines, and IFN- I	IRI, RA, cancer, and other inflammatory diseases
		Ubiquitous, highly expressed in epithelial cells, DCs, monocytes, macrophages. T cells, B cells, and NK cells	Cytoplasmic DNA	cGAS, AIM2	Promotes the production of IFN- 1, IL-1β, and IL-18 secretion and initiates pyroptosis, and other cytokines and chemokines	Cancer chronic kidney disease

**Table 2:** Toll-like receptors and their expressing cells and ligands.

TLRS	Cell type	Exogenous ligand
TLR1	Monocyte/macrophage, neutrophils, B cells	Lipoprotein
TLR2	Monocyte/macrophage, neutrophils	LTA, peptidoglycan, lipoproteins and zymosan
TLR3	B cells and T cells	Viral dsRNA
TLR4	Monocyte/macrophage, neutrophils, mast cells, B cells	Lipopolysaccharide, viral envelope protein, and viral fusion protein
TLR5	Monocyte/macrophage, neutrophils,	Flagellin
TLR6	Monocyte/macrophage, neutrophils, B cells	LTA, lipoproteins, and zymosan
TLR7	Monocyte/macrophage, neutrophils, cells	Viral ssRNA, synthetic antiviral compounds
TLR8	Monocyte/macrophage, neutrophils, mast cells	Viral ssRNA, synthetic antiviral compounds
TLR9	Monocyte/macrophage, neutrophils, B cells, T cells	Bacterial and viral DNA
TLR10	Monocyte/macrophage, B cells	Triacylated lipopeptides

## Data Availability

The data that support the findings of this study are available from the corresponding author upon reasonable request.

## Nomenclature

SI: Sterile inflammation
DAMPs: Damage-associated molecular patterns
PAMPs: Pathogen-associated molecular patterns
PRRs: Pattern recognition receptors
HMGB1: High mobility group box 1
vWF: von Willebrand factor
NAFLD: Nonalcoholic fatty liver diseases
TLR: Toll-like receptor
NLR: Nod-like receptor.
